# Effect of Welding Gap of Thin Plate Butt Welds on Inherent Strain and Welding Deformation of a Large Complex Box Structure

**DOI:** 10.3390/ma17091934

**Published:** 2024-04-23

**Authors:** Liping Zhang, Genchen Peng, Fan Yang, Zhengyu Meng, Xiaoming Yuan, Yangyang Fan, Wen Li, Lijie Zhang

**Affiliations:** 1 Hebei Key Laboratory of Heavy Machinery Fluid Power Transmission and Control, Yanshan University, Qinhuangdao 066004, China; 15094347413@163.com (L.Z.); xiaomingbingbing@163.com (X.Y.); liwen9933@126.com (W.L.); 2 Jiangsu XCMG Construction Machinery Research Institution Ltd., Xuzhou 221000, China; 17824830980@163.com (F.Y.); 15543156312@163.com (Z.M.); 3 State Key Laboratory of Advanced Welding and Joining, Harbin Institute of Technology, Harbin 150001, China; fanyangyang@hit.edu.cn; 4 Zhengzhou Research Institute, Harbin Institute of Technology, Zhengzhou 450018, China

**Keywords:** laser welding 304L stainless steel, FEM simulation, welding deformation, inherent strain, complex structure

## Abstract

In this study, an effective numerical model was developed for the calculation of the deformation of laser-welded 3 mm 304L stainless steel plates with different gaps (0.2 mm, 0.5 mm, and 1.0 mm). The welding deformation would become larger when the welding gaps increased, and the largest deformation values along the Z direction, of 4 mm, were produced when the gap value was 1.0 mm. A larger plastic strain region was generated in the location near the weld seam, since higher plastic deformation had occurred. In addition, the tensile stress model was also applied at the plastic strain zone and demonstrated that a larger welding gap led to a wider residual stress area. Based on the above results, inherent deformations for butt and corner joints were calculated according to inherent strain theory, and the welding formation for the complex structure was calculated with different gaps. The numerical results demonstrated that a larger deformation was also produced with a larger welding gap and that it could reach the highest value of 10.1 mm. This proves that a smaller welding gap should be adopted during the laser welding of complex structures to avoid excessive welding deformation.

## 1. Introduction

304L stainless steel has always been excellent when utilized in the fabrication of large components in the fields of petrochemicals, machine tools, nuclear, medical applications, and power industries, due to its excellent resistance to corrosion, good formability, and stable mechanical properties under high temperature [1,2,3,4,5,6,7]. During the fabrication of large structural components, many different structures with various shapes should be connected, and the employment of welding technology becomes necessary.

At present, different welding technologies are adopted in various circumstances. For instance, Yan et al. obtained arc-welded 304 stainless steel joints from TIG technology [8]. Arc welding has a large heating area and proved to be able to weld samples with large gaps. A laser-arc hybrid could also be adopted for the welding of 304L stainless steel. This welding technology demonstrated good penetration and a higher welding speed than that of single-arc technology [9,10]. Nevertheless, the assembling process of that experimental system was relatively complex, and many further trial experiments should be conducted to obtain the optimized couplings [11]. Compared to this welding technology, single-laser welding technology has become more and more popular in the welding of thin 304L stainless steel since it has the advantages of higher welding speed, an accurate heating area, and smaller welding deformation [12,13,14,15]. For instance, Abdul et al. successfully joined 304L stainless steel with laser technology, employing a welding speed of 1.5 m/min [16]. They found that the heating effect zone was only 1.15 mm.

During laser welding, gaps are produced in the butt joint due to assembling errors and irregular edges. The presence of the gap will eventually affect the weld formation [17,18]. For instance, Webster discovered that different welding gaps produce different melting volumes at the bottom of the weld seam, because more molten materials filled up that section and, finally, led to a different welding deformation during laser-arc hybrid welding [19]. Xia et al. discovered that a welding seam with different melting widths between the middle and top regions was obtained with different gaps during the laser-arc hybrid welding of Q235 steel [20]. This phenomenon produces various deformation behaviors in joints. Therefore, it is necessary to know the welding formation and deformation values for the laser welding of 304L stainless steel joints with different gaps.

To avoid repeated experiments and to reduce costs, the finite element method (FEM) can be adopted [21], and many researchers have made investigations into the numerical simulation of laser welding deformation. Belhadj et al. developed a numerical model for the fusion zone of laser welded magnesium alloys [22]. Belitzki et al. developed a numerical model to predict the welding deformation of laser-welded joints [23]. They also combined numerical simulation with artificial intelligence to obtain an optimized welding sequence, which proved to be beneficial to the reduction of welding deformations. For the prediction of large structures, the thermal-plastic-elastic method is hard to carry out due to extensive meshing and a long calculation duration; therefore, the inherent strain method is an effective alternative. Li et al. predicted the deformation of arc-welded ship block subassemblies with dimensions of 1810 mm, 1070 mm, and 500 mm [24]. They compared the calculated duration between inherent strain and the FEM model. The calculated durations for FEM and the inherent strain method were 20 h and 3 min, respectively. The calculated results, according to the inherent strain method, demonstrated a maximum relative deviation of 9.9% compared with experimental values. Murakawa et al. calculated the deformation of arc-welded thin plate structures with dimensions of 4000 m, 2000 m, and 300 mm, using the inherent strain method [25]. They also optimized the welding sequence to obtain a smaller welding deformation.

The aim of this research was to investigate the welding formations of laser-welded 304L stainless steel with different gaps (0.2 mm, 0.5 mm, and 1.0 mm). Then, the FEM model was developed to calculate the welding deformation, plastic strain, and residual stress of these three joints. This research hopes to give guidance regarding parameter optimizations in the practical laser welding of 304L stainless steel.

## 2. Experimental and Numerical Procedures

### 2.1. Selected Materials and Experimental Equipment

**Figure 1:**
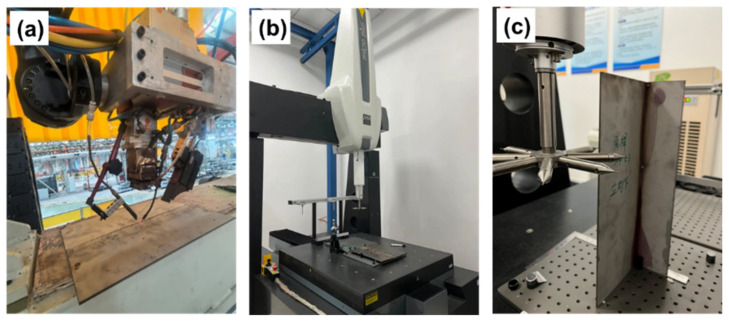
Laser welding of 304L stainless steel and measurement of deformation: (**a**) Laser welding system, (**b**,**c**) Measurement of welding deformation by three-coordinate measurement.

In this research, the IPG YLR-10000 fiber laser (Oxford, MA, USA) and KUKA robot (Augsburg, Germany) were assembled into a laser welding system. 304L stainless steel was selected as the base material. Chemical compositions are presented in Table 1. The dimensions for the base material were 400 mm^L^ × 300 mm^W^ × 3.0 mm^T^. During laser welding, 308L stainless steel filler with a diameter of 1.0 mm was adopted as the filling material. The 308L filler recorded a tensile strength of 614 MPa and an elongation of 38%.The laser welding process was presented in Figure 1a.

The laser was focused on the base metals and vertically irradiated on the workpiece. Before the welding process, the base metals were cleaned by a chemical agent and polished to remove surficial oxidation, in order to obtain a satisfactory welding formation [26]. To protect the oxidation of the weld seam, Argon protective gas was flowed behind the molten pool to obtain a satisfactory welding formation. The welding process is listed in Table 2.

When the welding experiment was complete, the samples were prepared for optical micrograph (OM, OLMPUSeDSX510, Olympus, Tokyo, Japan) observation. The prepared sample was 15 mm long and 5 mm wide, with a thickness of 3 mm. The preparation processes for samples for OM observation were as follows: samples were ground using #50 (ANSI standard), #100, #200, #400, #800, #1200, and #1200 sandpaper, in sequential order. Then, these ground samples were further refined and polished with diamond paste with a diameter of 0.5 μm and 0.25 μm. Then, the ground samples were flushed in sequential order by water and acetone and, finally, were dried. After the above polishing process, a sample with a mirror-like surface was obtained for further observation. OM was adopted to observe the morphology of the weld profile (containing fusion zone and base material).

The welding deformations of these samples, obtained with different gaps, were measured by three-coordinate measurement, which was performed on the Hexagon Micro Plus 10.12.08 (Aarau, Switzerland). According to the above method, welding profile and deformation could be obtained, and these results were utilized to verify the numerical model. The measuring process was presented as seen in Figure 1b,c.

### 2.2. Numerical Simulation

**Figure 2:**
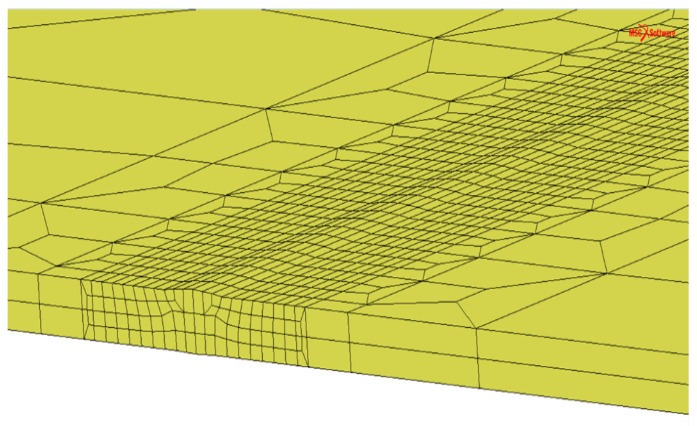
Generated model for different joints.

**Figure 3:**
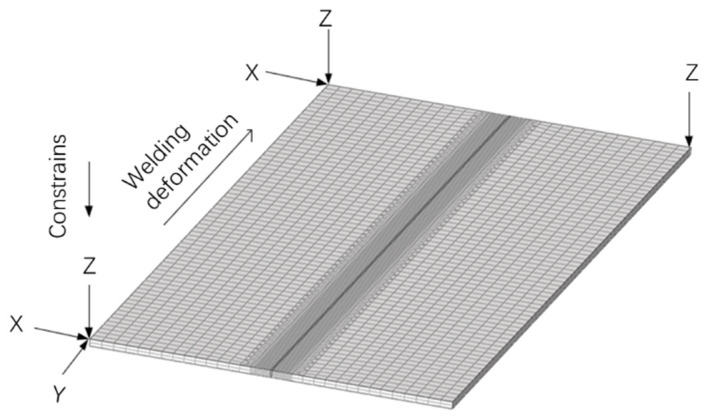
Mechanical boundary condition for simulation models.

To calculate the deformation of the welded samples, a numerical model with dimensions of 300 mm^L^ × 150 mm^W^ × 4.0 mm^T^ was developed, as seen in Figure 2. To ensure accuracy and to shorten the calculation duration of the simulation, coarse and fine meshes were divided between the interior (coarse) and outer region (fine) of the weld seam [27], as seen in Figure 3. In this research, traditional six-node constraints were adopted, as seen in Figure 3. The thermo-mechanical properties of 304L stainless steel are listed in Table 3. The welding deformation was calculated by the FEM software, JWRIAN (V103), which was developed by Osaka University.

In this research, the laser absorption coefficient was set as 0.8. The heat source for the laser energy is described by Gaussian equations [28,29]:

(1) $$q_{laser}(x,y,z)=6\sqrt{3}\frac{\eta_{laser}P_{laser}}{\pi \sqrt{\pi }r_{laser}^{3}}\exp\left(-\frac{3[{\left(x-x_{laser}\right)}^{2}+{\left(y-y_{laser}\right)}^{2}+{\left(z-z_{laser}\right)}^{2}]}{r_{laser}^{2}}\right)$$

In Equation (1), *P_laser_* represents laser power; *η_laser_*, laser energy absorption efficiency, *r_laser_*, the radius of laser spot; and *x_laser_*, *y_laser_* and *z_laser_* represent the center coordinates of the laser heating source along the *x*, *y*, and *z* directions, respectively. In this research, the absorption coefficient *η_laser_* was set as 0.85, and the laser spot diameter was 0.6 mm. The movement of the heating source was controlled by a subroutine programmed in Fortran.

## 3. Results and Discussion

### 3.1. Verification of Developed Models

**Figure 4:**
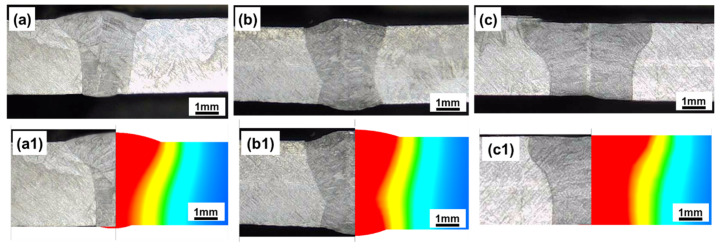
Comparisons for the numerical and experimental fusion lines. (**a**,**a1**) 0.2 mm, (**b**,**b1**) 0.5 mm, (**c**,**c1**) 1.0 mm.

To illustrate the accuracy of the developed models, Figure 4 compares the numerical and experimental fusion lines to present the corresponding results. As seen in Figure 4, a strong correlation in the numerical and experimental results was found, which indicated that the developed model in this research was effective. In Figure 4, the different colors in the model represent different zone temperatures.

### 3.2. Welding Deformations

**Figure 5:**
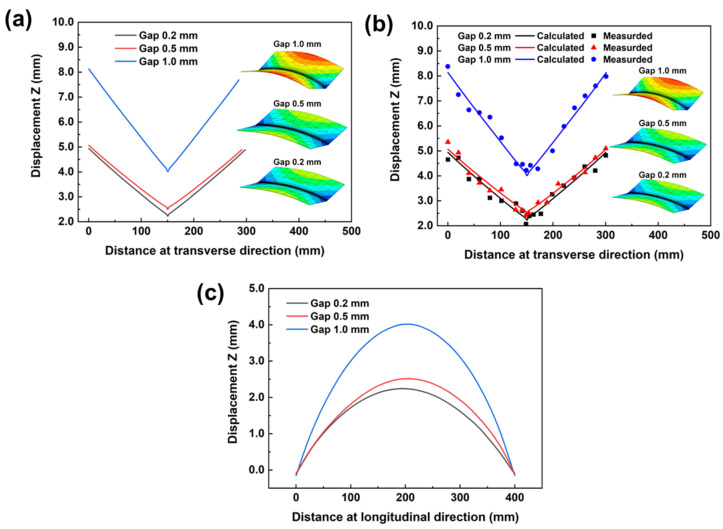
Comparisons for Displacement Z for different joints. (**a**) Displacement Z along transverse direction, (**b**) Comparisons for experimental and numerical Displacement Z along transverse direction, (**c**) Displacement Z along longitudinal direction.

During laser welding, deflection along the Z direction (Displacement Z) was the most important deformation parameter, and its value had great influence on final joint strength [31]; therefore, Displacement Z was calculated, and the corresponding results are presented in Figure 5. As seen in Figure 5a, it was found that Displacement Z along the transverse direction would become smaller with each reduction in gap distance. The highest value for Displacement Z, of 4.0 mm, was produced at the center of joints with a 1.0 mm gap, and the lowest value, of 2.2 mm, was recorded for joints with a 0.2 mm gap. As reported in a previous publication [20], a larger melting zone induces larger welding deformations. According to the fusion zone, as presented in Figure 3, the largest melting zone was produced when a 1.0 mm gap was adopted, which finally resulted in the largest Displacement Z.

From the above observed and analyzed results, it was found that gap distance had a great effect on the final welding deformation. When gap distances were 0.2 and 0.5 mm, the deformation values were similar; however, the values expanded quickly when the gap distance was 1.0 mm. Therefore, it can be concluded that a lower gap distance should be adopted during the laser welding of 304L stainless steel, and the gap distance should be less than 0.5 mm. It should be noted that deformation was different on the two sides of the sample; this was caused by the constraint difference, as seen in Figure 3.

**Figure 6:**
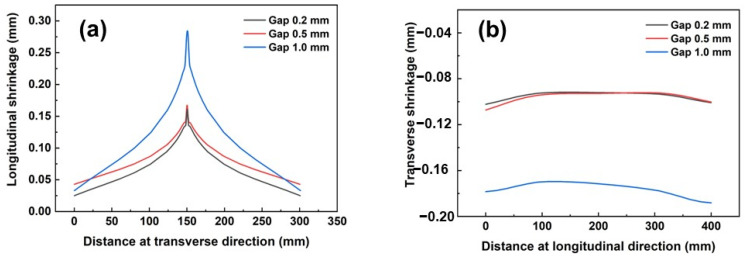
Comparisons for Displacement Z for different joints. (**a**) Longitudinal shrinkage along the transverse direction, (**b**) Longitudinal shrinkage along the longitudinal direction.

The shrinkage deformation for three joints is presented in Figure 6, which illustrates the finding that the shrinkage deformation produced in the joint with a 1.0 mm gap was much larger than in the other two joints. In line with previous reporting [32], shrinkage deformation during laser welding was caused by the volume of liquid metal. A lager volume of liquid metal led to larger shrinkage. This result could also be proved by metallography, as seen in Figure 3. It was immediately apparent that the volume of liquid metal was greater for the joint produced with a 1.0 mm gap.

### 3.3. Plastic Strain

**Figure 7:**
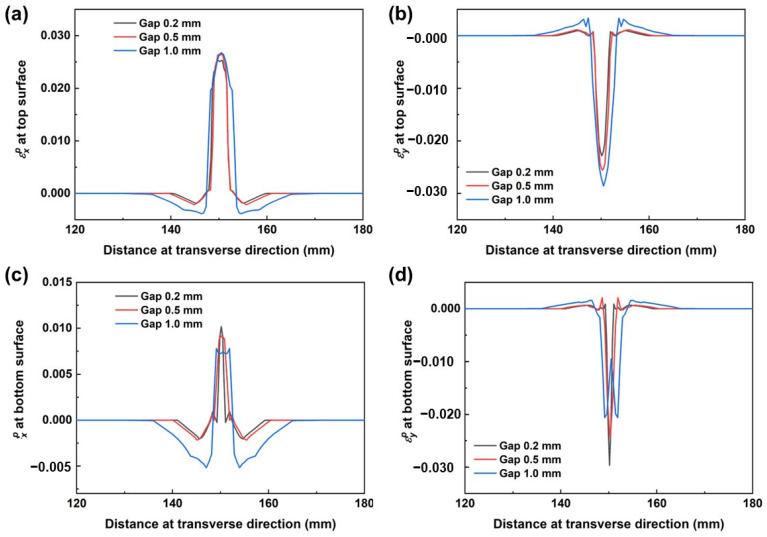
Comparisons of plastic strain along transverse direction for different joints at top and bottom surfaces. (**a**) Transverse plastic strain along top surface, (**b**) Longitudinal plastic strain along top surface, (**c**) Transverse plastic strain along bottom surface, (**d**) Longitudinal plastic strain along bottom surface.

To provide a clearer observation of the impact of welding gaps on plastic strain, longitudinal and transverse plastic strains (ε_p_^x^ and ε_p_^y^) along the surfaces of models were extracted, as illustrated in Figure 7a,b. At the same time, the values of ε_p_^x^ and ε_p_^y^, along the bottom surface of the model, were also extracted and are illustrated in Figure 7c,d.

As seen in Figure 7, it was found that larger plastic strain values were present in the fusion zone and heat-affected zone, exhibiting a similar variation tendency to shrinkage. Little difference was recorded in the transverse plastic strain along the top surface, as seen in Figure 7a. For the transverse plastic strain ε_p_^y^ along the bottom surface, the largest values were produced when the gap distance was 1.0 mm. Plastic strain ε_p_^x^ was caused by the fusion liquid metal. According to Figure 4, the widest fusion line at the bottom surface was generated when the gap distance was 1.0 mm, which induced the largest plastic strain. Comparing values for ε_p_^x^ and ε_p_^y^, an interesting phenomenon was discovered: ε_p_^y^ was larger than that of ε_p_^x^. This confirms results published by Ma et al. [33], who have proposed that this phenomenon is caused by weaker constraints during the laser welding process, as seen in Figure 3.

### 3.4. Residual Stress

**Figure 8:**
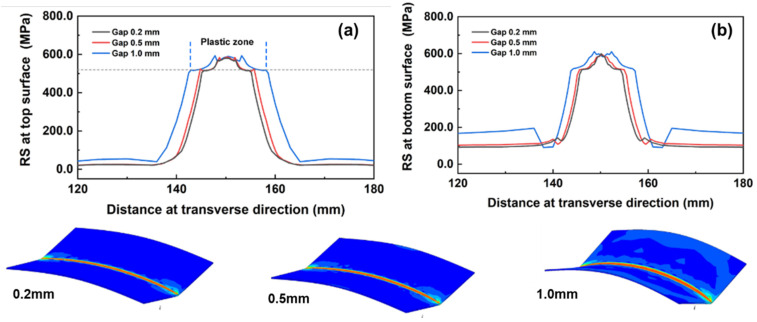
Von Mises stress distribution for different joints at top and bottom surfaces. (**a**) Residual stress along top surface, (**b**) Residual stress along bottom surface.

The residual stress for three joints was also calculated, and the corresponding results are presented in Figure 8. Figure 8 illustrates that the largest Von Mises stress value level and the widest plastic strain area were both produced at the joint with a 1.0 mm gap distance. This finding aligns with previous reporting [34].

When molten pool was cooled, a material property difference was produced between the fusion zone and basic metals, which induced the generation of residual stress. At this point, differences in the material properties of the fusion zone and base metals were produced. As seen in Figure 5, the joint produced with a 1.0 mm gap demonstrated the largest fusion width in both top and bottom regions. When the welding process was finished, a larger material property difference was produced. During the cooling process, partial elastic strain was recovered, leaving only plastic strain behind. Then, the largest Von Mises stress value level and the widest plastic strain area were both able to be generated, as seen in Figure 8a,b. Under this condition, a larger welding deformation was also produced, as seen in Figure 5. An interesting phenomenon was found when observing the values of SX: SX peak values exceeded the yield strength of 304L stainless steel, and this was caused by the strain hardening.

### 3.5. Inherent Deformation

**Figure 9:**
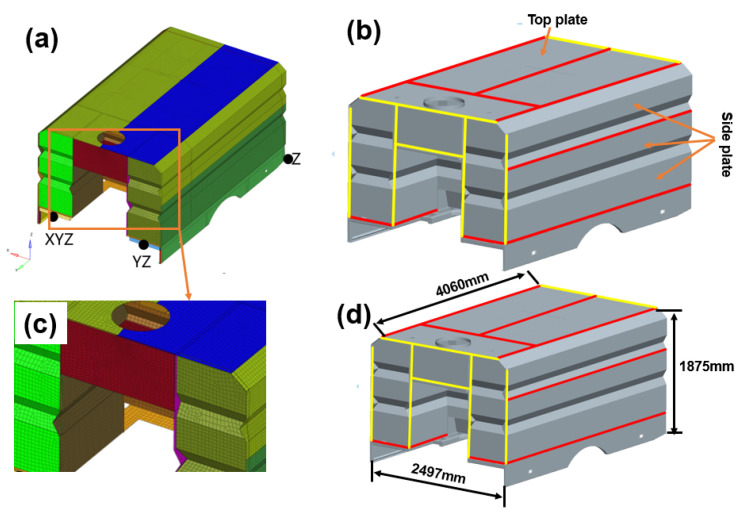
Calculated complex box structure. (**a**) Generated meshes and constraints, (**b**) Welding seam, (**c**) Amplification for generation of meshes, (**d**) Dimension for structure.

To calculate the final deformation of the structure, the inherent strain method was adopted. Compared to the thermal-plastic-elastic method, the inherent strain method can predict welding deformation in a far shorter duration with acceptable accuracy. In this method, four basic parameters are calculated, as presented in Equations (2)–(5): longitudinal inherent shrinkage $\delta_{x}^{*}$, transverse inherent shrinkage $\delta_{y}^{*}$, longitudinal inherent bending $R_{x}^{*}$, and transverse inherent bending $R_{y}^{*}$ [35]. The calculated results are presented in Table 4. When these four inherent deformation parameters were obtained, the welding deformations for the complex box structure with multiple welding lines, as seen in Figure 9, were predicted. Additionally, inherent deformations for corner joints were also calculated, since the fabrication of large complex box structure contains corner joints, and the corresponding results are listed in Table 5. The dimensions of the complex box structure were 4060 mm (length) × 2497 mm (width) × 1875 mm (height), as seen in Figure 9d.

As demonstrated in the inherent deformation values, it was found that the transverse deformation was an order of magnitude higher than the longitude deformations. This has proved that the welding deformation was mainly determined by the transverse deformation.

(2) $$\delta_{x}^{*}=\frac{1}{h}\iint \varepsilon_{x}^{p}dydz$$

(3) $$\delta_{y}^{*}=\frac{1}{h}\iint \varepsilon_{y}^{p}dydz$$

(4) $$R_{x}^{*}=\frac{1}{I}\int \varepsilon_{x}^{p}(z-\frac{h}{2})dydz$$

(5) $$R_{y}^{*}=\frac{1}{I}\int \varepsilon_{y}^{p}(z-\frac{h}{2})dydz$$

**Figure 10:**
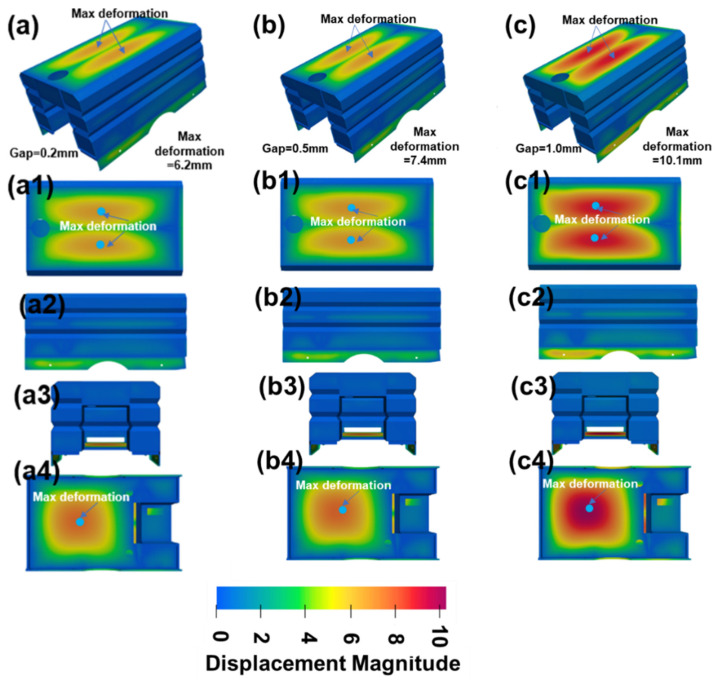
Welding deformation for structure with different welding gaps. (**a**–**a4**) 0.2 mm, (**b**–**b4**) 0.5 mm, (**c**–**c4**) 1.0 mm.

Before the final calculation, some simplifications were implemented: (1) the generated meshes for the model were shells, comprising 75,732 nodes and 782,999 elements. The dimension for the meshes were 35 mm × 35 mm, as seen in Figure 9c. (2) The restrained conditions are shown in Figure 10a. A traditional six-node constraint was adopted. (3) Various structures with little difference in thickness were treated as the same. Under these amplifications, the calculated duration could be shortened and accuracy maintained. For this structure, a total of 24 weld seams were present, which included 10 butt weld seams and 14 corner weld seams.

The calculated results are shown in Figure 10. A similar deformation tendency was discovered, as seen in Figure 10a–c. A larger red area is produced with larger welding gaps, which indicates a larger welding deformation. For the deformation distribution, smaller values were produced at the side structures, while larger values were produced at the cover plates, as seen in Figure 10a–c. It was found that the maximum deformation was generated at the upper cover plate. The maximum deformation for each of the three structures was 6.2 mm, 7.4 mm, and 10.1 mm, when the welding gaps were 0.2 mm, 0.5 mm, and 1.0 mm, respectively. This proved that a larger welding gap was disadvantageous for the control of the final welding deformations.

## 4. Conclusions

In this research, a satisfactory numerical model was developed to predict the welding deformation of a laser-welded 304L stainless steel butt joint (with welding gaps of 0.2 mm, 0.5 mm, and 1.0 mm). Based on the calculated results, the deformation tendency of a complex structure was calculated according to the inherent strain method. The conclusions are as follows:

1. Larger deformation values were produced with larger welding gaps (as expected), and the highest value for a welding deformation along the Z direction, of 4.0 mm, was generated with welding gaps of 1.0 mm, which was proportional with the volume of liquid metals. Welding deformation along the Z direction reduced approximately 45%, to 2.2 mm, when the welding gap was 0.2 mm.
2. A wider plastic strain region and larger welding residual stress value was recorded with larger gaps, since a larger fusion zone and greater deformation were produced. The highest tensile stress around the weld seam was larger than the yield strength of the base metal, since a strain-hardening phenomenon had occurred.
3. Inherent strain theory was adopted to calculate the deformation inherent to different gaps, and larger inherent deformations were produced with laser welding gaps. From the calculated results of the inherent deformation, it was found that transverse deformation was much greater than longitude deformation. This indicated that transverse deformation was the main deformation mechanism.
4. Welding deformations for complex box structures were calculated based on the calculated inherent deformations in different gaps. The highest deformations, of 10.1 mm, 7.4 mm, and 6.2 mm, were produced at the upper cover plate with a welding gap of 1.0 mm, 0.5 mm, and 0.2 mm, respectively. A maximum 30% displacement reduction occurred when a 0.2 mm gap was adopted, compared to a 1.0 mm gap. These findings suggest that a smaller welding gap should be adopted when welding complex structures in order to reduce welding deformation.

## Figures and Tables

**Table 1:** Chemical compositions and tensile strengths of base metals.

	Mn	Si	S	C	P	Cr	Ni	Zn	Cu	Fe	σ (UTS, MPa)
304L	1.50	0.60	0.015	0.030	0.020	19.0	9.0	0.05	0.05	Bal.	590
308L Filler	1.80	0.60	0.008	0.028	0.015	20.0	10.0	0.03	–	Bal.	614

**Table 2:** Laser welding parameters for 304L stainless steel.

Parameters	Values
Laser power (kW)	2.00
Welding speed (m/min)	1.2
Filler feeding speed (m/min)	2.0 m
Oscillated frequency (Hz)	50
Oscillated path	Linear
Oscillated amplitude (mm)	3
Gas flow rate (L/min)	15

**Table 3:** Laser welding parameters for 304L stainless steel [30].

Temperature (°C)	Density (kg/m^3^)	Specific Heat (J/kg/°C)	Conductivity (J/mm/°C)	Yield Strength (MPa)	Thermal Expansion Coefficient (/°C)	Young’s Modulus (GPa)
20	7884	459	0.014	264	1.69 × 10^−5^	197
100	7884	492	0.014	217	1.73 × 10^−5^	192
200	7802	510	0.015	186	1.80 × 10^−5^	184
300	7802	523	0.017	170	1.86 × 10^−5^	175
400	7781	539	0.018	155	1.91 × 10^−5^	166
600	7648	576	0.020	148	1.96 × 10^−5^	159
800	7525	603	0.023	91	2.02 × 10^−5^	151
1000	7402	639	0.027	58	2.05 × 10^−5^	105
1300	7269	690	0.033	20	2.11 × 10^−5^	20
1500	7249	699	0.120	9	2.16 × 10^−5^	9

**Table 4:** Inherent deformation of hybrid welded flat butt joints.

Initial Gaps	Longitudinal Inherent Shrinkage (mm)	Transverse Inherent Shrinkage (mm)	Longitudinal Inherent Bending (rad.)	Transverse Inherent Bending (rad.)
0.2 mm	0.00268	0.020246	−0.0006	0.033987
0.5 mm	0.00403	0.017627	−0.0007	0.031678
1.0 mm	0.01406	0.04980	−0.0009	0.053149

**Table 5:** Inherent deformation of hybrid welded corner joints.

Initial Gaps	Longitudinal Inherent Shrinkage (mm)	Transverse Inherent Shrinkage (mm)	Longitudinal Inherent Bending (rad.)	Transverse Inherent Bending (rad.)
0.2 mm	0.00476	0.1395	0	0.0082
0.5 mm	0.00556	0.1707	0	0.0073
1.0 mm	0.00686	0.2516	0	0.0054

## Data Availability

Data are contained within the article.
